# Effects of ketogenic diet on muscle mass, strength, aerobic metabolic capacity, and endurance in adults: a systematic review and meta-analysis

**DOI:** 10.1186/s41043-025-01090-z

**Published:** 2025-10-01

**Authors:** Yaqi Wang, Quanzhou Xiao, Zhenming Zhang, Yan Yang

**Affiliations:** 1 Metabolic Syndrome Research Center, Department of Metabolism and Endocrinology,, The Second Xiangya Hospital of Central South University, Changsha, 410011 Hunan China; 2 People’s Hospital of Ningxiang City, Ningxiang, 410600 Hunan China; 3 Department of Spine Surgery, The Second Xiangya Hospital, Central South University, Changsha, 410011 Hunan China

**Keywords:** Muscle mass, Muscle strength, Aerobic metabolic capacity, Endurance, Fat oxidation, Weight loss

## Abstract

**Background:**

The ketogenic diet (KD) has gained popularity as an efficient approach to weight loss and body fat loss. Concerns about reducing muscle mass and performance have been rising during weight loss, as muscle mass and functionality are crucial for health. However, the effects of the KD on muscles not only for athletes or trainers but also for adults with less physical exercise are still controversial.

**Methods:**

We conducted a thorough search of databases including Embase, PubMed, Cochrane Library, and Web of Science up to July 19, 2025. Three aspects of muscle assessment were conducted, including muscle mass, power and strength, aerobic metabolic capacity, and endurance. We included randomized and non-randomized controlled studies that compared the KD with other dietary interventions. Studies without control groups were excluded. A random-effects model would be utilized when significant differences in populations and interventions across studies were of concern. The GRADE system was employed to assess evidence quality, while evidence reliability was gauged via sensitivity analysis.

**Results:**

A total of 33 studies were analyzed, revealing no significant differences between the KD and other diets in muscle mass (WMD: 0.06, 95%CI: -1.97 to 2.09, *p* = 0.95), muscle power (countermovement jump: SMD: -0.06, 95%CI: -0.49 to 0.38, *p* = 0.80) and strength (squat: SMD: -0.19, 95%CI: -0.53 to 0.15, *p* = 0.27; bench press: SMD: -0.15, 95%CI: -0.49 to 0.18, *p* = 0.37). However, a significant decrease in fat-free mass (WMD: -0.48, 95%CI: -0.73 to -0.23, *p* < 0.001) and fat mass (WMD: -1.31, 95%CI: -2.06 to -0.57, *p* < 0.001) was observed in the KD group compared with the control group. The KD also improved fat oxidation (WMD: 0.13, 95%CI: 0.08 to 0.17, *p* < 0.001) and reduced respiratory exchange ratio (WMD: -0.07, 95%CI: -0.11 to -0.03, *p* < 0.001) during an exercise test. The VO_2max_ and VO_2max_ relative to body weight, treadmill time to exhaustion, and rating of perceived exertion were not significantly affected by KD.

**Conclusions:**

Among adult populations, KD can increase fat oxidation to modify muscle metabolism, while no significant reduction in muscle mass or strength was observed. Additional well-designed randomized controlled trials are warranted to definitively determine the effects of ketogenic diets on muscle parameters.

**Supplementary Information:**

The online version contains supplementary material available at 10.1186/s41043-025-01090-z.

## Highlights

The ketogenic diet is widely recognized for its weight loss effects, and this study provides evidence of its effect on muscle, including muscle mass, strength, aerobic metabolic capacity, and endurance.

The ketogenic diet does not have adverse effects on muscle mass or muscle strength.

The ketogenic diet enhances fat oxidation and reduces respiratory exchange ratio during treadmill tests, indicating a shift in muscle energy metabolism.

**Supplementary Information:**

The online version contains supplementary material available at 10.1186/s41043-025-01090-z.

## Introduction

Dietary intervention has historically been employed as a therapeutic approach for managing neurological diseases and extending lifespan [1, 2]. The global rise in obesity rates, attributed to shifts in lifestyle, has been substantial. The World Obesity Atlas 2024 predicts that overweight and obesity may affect more than 3.3 billion adults by 2035, up from more than 2.2 billion in 2020 [3]. Dietary strategies are the first-line approach for weight management and metabolic health improvement, with the ketogenic diet (KD) characterized by significant carbohydrate restriction, leading to increased fat and/or protein intake to induce a state of ketosis [4]. Typically, carbohydrate intake must be reduced below approximately 50 g per day or 10% of total caloric intake for several days to achieve ketosis [5–7]. Research has demonstrated that this diet not only leads to weight loss but also results in a significant reduction in body fat [8].

Various weight loss methods, including dietary restrictions, can lead to a decrease in muscle mass in addition to fat loss [9–11]. Given the significant impact of muscle mass and performance on health outcomes, particularly in relation to aging and metabolic disorders [12] the preservation or enhancement of muscle while reducing fat has emerged as a critical focus. A diet high in fats and low in carbohydrates, potentially reducing muscle protein breakdown through decreased leucine oxidation, can help preserve muscle mass during weight loss [13, 14].

The KD may affect both caloric and carbohydrate restriction, consequently influencing the metabolic pattern of muscle, including attenuating glucose utilization and anti-lipolytic effects [15]. Ketone bodies can serve as an alternative energy substrate for skeletal muscle, particularly during carbohydrate restriction or prolonged fasting [16]. Emerging evidence suggests that the KD may promote mitochondrial biogenesis and improve mitochondrial function in skeletal muscle [17]. In addition to their traditional role in energy metabolism, ketone bodies have been proposed to act as signaling molecules [18]. Some studies suggest that ketone bodies may support muscle cell proliferation [19] and may also contribute to enhanced antioxidant capacity, activation of autophagy, and anti-inflammatory responses—all of which could potentially influence muscle mass and performance [20]. Several studies summarized the influence of the KD on muscle performance in athletes or trained adults [21, 22]. Whether the effects of the KD extend to untrained individuals remains unclear. This study aimed to systematically evaluate the effects of the KD on muscle-related outcomes, including muscle mass, strength, aerobic capacity, and endurance performance, in populations with or without exercise training.

## Methods

### Search strategy

The analysis methodologies and eligibility criteria were pre-planned, and documented in a PROSPERO-registered protocol (CRD42024516932).

We selected Embase, PubMed, Cochrane Library, and Web of Science published up to July 19, 2025, utilizing a comprehensive list of keywords related to various types of ketogenic diets and exercise outcomes. (“Ketogenic” OR “KD” OR “VLCKD” OR “Very low carbohydrate ketogenic diet” OR “High fat diet” OR “HFD” OR “Low carbohydrate diet” OR “LCD” OR “Very low calorie ketogenic diet” OR “classic ketogenic diet” OR “Long-chain triglyceride diet” OR “LCT diet” OR “Long chain triglyceride diet” OR “Low glycemic index treatment” OR “Medium chain triglyceride ketogenic diet” OR “LGIT” OR “MCT diet” OR “Modified Atkins diet”) AND (“strength” OR “force” OR “hypertrophy” OR “muscle mass” OR “hypertrophic response” OR “endurance” OR “aerobic*”) AND (“randomized controlled trial” OR random* OR placebo[Title/Abstract]). All retrieval were completed within a week. A manual search also was accomplished according to the references of critical articles published, which served as the origin for other sources.

### Study selection

The criteria for inclusion were as follows: (1) full text was available; (2) the dietary intervention had to involve KD (or a variation of KD) and a control group (any other dietary intervention), the low-carbohydrate, high-fat (LCHF) diet with less than 10% carbohydrates is considered a KD; (3) belonging to one of the following studies: observational, experimental (peer-reviewed), quasi-experimental or empirical (i.e., not a letter, review, meeting proceedings, or case series); (4) evaluation of muscle outcomes including muscle mass, strength, aerobic metabolic capacity and endurance. Exclusion criteria were as follows: (1) inability to extract data separately; (2) all studies that supplemented another compound combined with KD in the intervention group; (3) participants who were not adults or any circumstances that could potentially influence the results; (4) non-human studies, including animal experiments.

Upon elimination of duplicate literature, two independent investigators (WYQ and XQZ) conducted the literature screening and revision. Initially, the titles and abstracts of the identified articles were reviewed for relevance. Subsequently, full-text articles that met the eligibility criteria were retrieved and selected for further analysis. Any disagreements were resolved through dialogue with a third reviewer (YY).

### Data extraction and quality assessment

Two reviewers conducted data extraction independently, focusing on the study population characteristics, intervention details, and muscle outcomes such as muscle mass, muscle power and strength, aerobic metabolic capacity and endurance. For studies with essential data missing, we attempted to get in touch with the corresponding author. The risk for bias was assessed by the reviewers following PRISMA recommendations, ensuring the reliability and validity of the research. For randomized controlled trials (RCTs) and crossover trials, the Cochrane risk of bias 2 tool (RoB 2) was utilized, while non-randomized studies of interventions (NRSI) used the Risk Of Bias In Non-randomized Studies-Of Interventions (ROBINS-I) to evaluate bias. The studies that include a control group but do not specify whether they are randomized are classified as intervention studies. The quality assessment of intervention studies used ROBINS-I to assess the risk of bias. Also, the quality of evidence was evaluated by using GRADEpro 3.6.

### Statistical analysis

The outcome data were extracted from the original study and expressed as mean ± SD. According to the Cochrane Handbook, if the original text does not provide the net change in outcome variables between the intervention group and the control group, one should calculate the mean and standard deviation before and after the intervention using the appropriate formula (Mean_change_= Mean_post_­Mean_pre_; SD_change_=$\:\sqrt{{SD}_{pre}^{2}+{SD}_{post}^{2}-2\cdot\:corr\cdot\:{SD}_{pre}\cdot\:{SD}_{post}}$). These values should then be substituted into the software to obtain the combined effect size. To reasonably estimate the correlation coefficient (Corr), sensitivity analysis utilized values of 0.5, 0.7, and 0.9. To assess publication bias, a funnel plot and Egger’s test were employed, with a *p*-value less than 0.05 indicating the probable bias. The standardized mean differences (SMDs) between pre- and post-intervention were calculated and weighted by inverse variances if units of measurement were not uniform. On the contrary, the weighted mean difference (WMD) would be calculated in the analysis. The combined effect test results were represented by the Z value, and the *P* value was obtained according to the Z value. *P* < 0.05 was considered statistically significant. Heterogeneity was measured by *I*² statistics and the Cochran Q test. A random-effects model would apply when the variation in true effect sizes across studies is due to differences in study populations, interventions, and methodologies. Subgroup analysis was based on the characteristics of participants, duration of intervention, and the study design. Sensitivity analyses involved systematically removing individual studies from the meta-analysis for assessment. The meta-analysis was conducted by using Review Manager 5.4.1 and STATA 13.0.

## Results

### Literature search

**Fig. 1:**
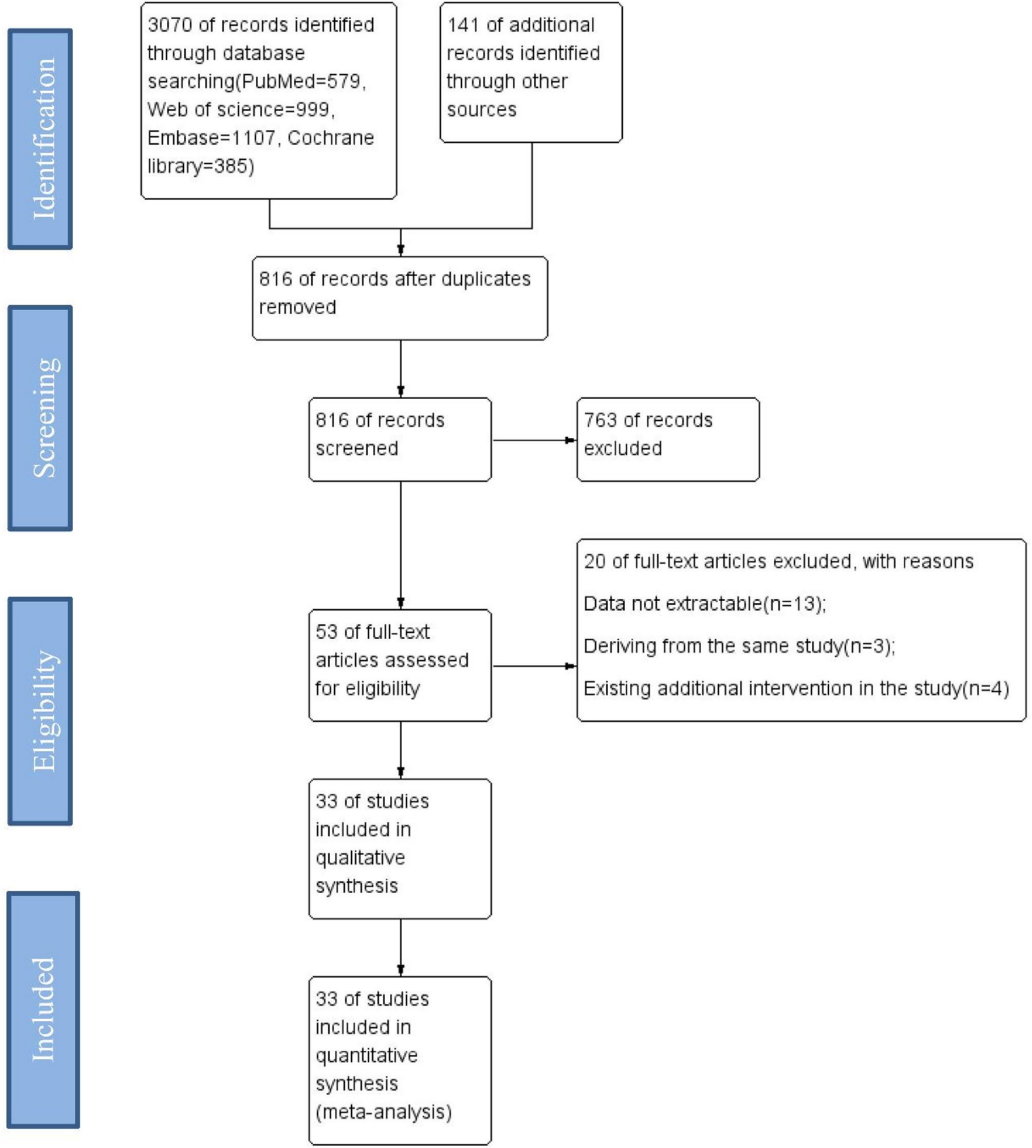
Flow diagram showing selection process

We totally yielded 3211 citations, of which 33 studies were enrolled in our following analysis. Initially, the search covered various approaches such as PubMed, Web of Science, Embase, Cochrane Library, and other sources, totaling 579, 999, 1107, 385, and 141 records, respectively. After eliminating duplicate records, screening titles and abstracts, and checking inclusion and exclusion criteria, we excluded 2395 records. We then thoroughly reviewed the full text of the 53 eligible reports, leading to the rejection of 20 reports due to various reasons: data not extractable(*n* = 13); deriving from the same study(*n* = 3); existing additional intervention in the study(*n* = 4) (Fig. 1).

### Characteristics of the included studies

The study participants of the 33 included studies varied in number, ranging from 7 to 115, with the invitation period varying from 2 weeks to 2 years. Thirty-two studies reported changes in body composition, specifically body mass, fat mass, and lean mass, while 5 of them also included muscle mass data. Among the included studies, 13 studies provided data on muscle power (countermovement jump (CMJ)) and strength (bench press, squat, handgrip strength, etc.), and 11 studies investigated the effect of KD on aerobic metabolic capacity and endurance (Table 1).

**Table 1:** Characteristics of included studies

Study ID	Subjects	Age (Years)	Study Design	Dietary Components	Intervention Duration	Body composition assessment	Muscle strength and power	Aerobic metabolic capacity and endurance
Greene, David A et al. 2018 [31]	14 competitive lifting athletes	34 ± 10.5	Randomized crossover study	LCKD:8.1 ± 2.0%CHO, 69.1 ± 5.6%fat, 22.9 ± 4.6%protein; UD:44.8 ± 4.8% CHO, 33.2 ± 6.0%fat, 22.0 ± 6.0%protein	3 months	body mass, fat mass, lean mass	Lifting performance	NA
Pavel Kysel et al. 2023 [32]	25 regular resistance/aerobic training males	CKD:23 ± 5; RD:24 ± 4	RCT	CKD: five-day low-carbohydrate diet in a week; RD:55%CHO, 30%fat, 15%protein	8 weeks	body mass, fat mass, fat-free mass	NA	NA
A. Antonio Paoli et al. 2021(a) [33]	16 semi-professional male soccer players	25.5 ± 2.8	RCT	KD:9 ± 3%CHO, 64 ± 3%fat, 28 ± 4%protein; WD:51 ± 4%CHO, 20 ± 8%fat, 28 ± 3%protein	30 days	body mass, fat mass, lean mass	CMJ, Yo-yo	NA
Salvador Vargas-Molina et al. 2020 [34]	21 strength-trained women	27.6 ± 4.0	RCT	KD:9.1 ± 1.3%CHO, 64.1 ± 2.3%fat, 26.8 ± 2.3%protein; NKD:57.0 ± 1.9%CHO, 23.3 ± 1.6%fat, 19.7 ± 1.4%protein	8 weeks	body mass, fat mass, fat-free mass	Squat, CMJ	NA
A. Antonio Paoli et al. 2021(b) [35]	19 competitive male body builders	27.42 ± 10.54	RCT	KD:5.00 ± 0.00%CHO, 68.00 ± 2.27%fat, 24.65 ± 1.24%protein; WD:55.00 ± 0.00%CHO, 19.97 ± 0.91%fat, 25.03 ± 0.91%protein	2 months	body mass, fat-free mass	Bench press, squat	NA
Salvador Vargas et al. 2018 [36]	24 overload trained men	30 ± 4.5	RCT	KD:<10%CHO, 70%fat, 20%protein; NKD:55%CHO, 25%fat, 20%protein	8 weeks	body mass, fat mass, lean mass	NA	NA
Hae-Ryeon Choi et al. 2018 [37]	46 adults with BMI > 25 kg/m^2^	KD:29.5 ± 9.0; BD:26.0 ± 7.5	RCT	KD:3%CHO, 90%fat, 7%protein; BD:54%CHO, 30%fat, 16%protein	2 weeks	body mass, fat mass, skeletal muscle mass	NA	NA
Vladimir Vidic et al. 2021 [38]	20 resistance-trained men	42.7 ± 1.5	RCT	KD:5%CHO, 75%fat, 20%protein; NKD:15%CHO, 65%fat, 20%protein	8 weeks	body mass, fat mass, lean mass	Bench press, squat	NA
Jacob M. Wilson et al. 2020 [39]	25 resistance-trained males	KD:23.5 ± 4.5; WD:21.3 ± 3.7	Randomized crossover study	KD:5%CHO, 75%fat, 20%protein; WD:55%CHO, 25%fat, 20%protein	10 weeks	body mass, fat mass, lean mass	Bench press, squat, wingate test	NA
Michael Vogt et al. 2003 [40]	11 male athletes	31.6 ± 2.0	Randomized crossover study	HFD:31.4 ± 0.7%CHO, 52.9 ± 0.7%fat, 14.4 ± 0.4%protein; LFD:68.2 ± 0.9%CHO, 16.5 ± 0.6%fat, 14.3 ± 0.8%protein	5 weeks	body mass, fat mass	NA	VO_2_; VO_2_ relative to body weight; VCO_2_
Thomas P. Wycherley et al. 2014 [41]	43 abdominal obesity adults with at least one metabolic risk factor	49.2 ± 1.1	RCT	HC:47.9 ± 0.8%CHO, 26.0 ± 1.0%fat, 23.7 ± 0.5%protein LC:7.7 ± 0.7%CHO, 57.2 ± 0.7%fat, 33.7 ± 0.4%protein	52 weeks	body mass, fat mass, fat-free mass	Handgrip strength	VO_2_; VO_2_ relative to body weight; fat oxidation; RER; TTE
Grant D. Brinkworth et al. 2009(a) [42]	60 overweight or obese adults with at least one metabolic risk factor	49.2 ± 1.2	RCT	LC:4%CHO, 61%fat, 35%protein; HC:46%CHO, 30%fat, 24%protein	8 weeks	body mass, fat mass, fat-free mass	Handgrip strength	VO_2_; VO_2_ relative to body weight; fat oxidation; RER; TTE; RPE
Philip J. Prins et al. 2019 [43]	7 competitive recreational distance male runners	35.6 ± 8.4	Randomized crossover study	LCHF:6.0 ± 1.3%CHO, 68.6 ± 2.1%fat, 25.1 ± 1.5%protein; HCLF:56.4 ± 2.6%CHO, 15.3 ± 1.1% protein, 27.8 ± 2.3% fat	6 weeks	body mass, fat mass, lean mass	NA	VO_2_; VO_2_ relative to body weight; VCO_2_; TTE; RER; fat oxidation
Tomas Dostal et al. 2019 [44]	24 moderately trained individuals	VLCHF:25.3 ± 2.0; HD:23.9 ± 3.8	Non-randomized parallel-group study	VLCHF:40 ± 6 g CHO, 149 ± 26 g fat, 113 ± 24 g protein; HD:201 ± 49 g CHO, 67 ± 16 g fat, 82 ± 30 g protein	12 weeks	body mass, fat mass, skeletal muscle mass	NA	VO_2_; VO_2_ relative to body weight; TTE; RER; RPE
Richard A. LaFountain et al. 2019 [45]	15 soldiers	KD:27.4 ± 6.8; MD:24.6 ± 9.0	Non-randomized study	NA	12 weeks	body mass, fat mass	Squat, CMJ, Bench press	NA
Fionn T. McSwiney et al. 2018 [46]	20 male endurance-trained athletes	LCKD:33.8 ± 6.9; HC:32.1 ± 6.4	Non-randomized study	LCKD:41.1 ± 13.3 g CHO, 259.3 ± 83.4 g fat, 130.7 ± 35.8 g protein; HD:400.3 ± 102.7 g CHO, 55.2 ± 10.7 g fat, 90.9 ± 23.6 g protein	12 weeks	body mass, fat mass, lean mass	100 km time trial, six second sprint, critical power test	VO_2_ relative to body weight
Karen M Skemp et al. 2021 [47]	20 resistance-trained women	20.27 ± 1.60	RCT	KD:10%CHO, 70%fat, 20%protein	4 weeks	body mass, fat mass, fat-free mass	NA	NA
Louise M. Burke et al. 2017 [26]	19 male race walkers	LCHF:28.3 ± 3.5; HCHO: 25.4 ± 4.0	Non-randomized study	LCHF:3.5%CHO, 78%fat, 17%protein; HCHO:60%CHO, 20%fat, 16%protein	3 weeks	body mass	NA	VO_2_; RER; RPE
Lukas Cipryan et al. 2018 [48]	18 moderately trained males	23.8 ± 2.1	Intervention Study	VLCHF:8 ± 3%CHO, 63 ± 13%fat, 29 ± 15%protein; HD: 48 ± 13%CHO, 35 ± 9%fat, 17 ± 3%protein	4 weeks	body mass, fat mass	NA	VO_2_; VO_2_ relative to body weight; TTE; RER; RPE; fat oxidation
Jesse Fleming et al. 2003 [49]	20 men	HFD:36 ± 12; CD:35 ± 13	Intervention Study	HFD:8 ± 3%CHO, 61 ± 4%fat, 30 ± 5%protein; CD:59 ± 7%CHO, 15 ± 1% protein, 25 ± 8% fat	6 weeks	NA	Wingate Power Output Data	VO_2_; VO_2_ relative to body weight; RER;
Maria Perticone et al. 2019 [50]	50 obese outpatients	46.8 ± 11.0	RCT	VLCKD:20%CHO, 20–30%fat, 50–60%protein; SHMD:55–60%CHO, 25–30%fat, 10–15%protein	12 months	body mass, fat mass, fat-free mass, muscle mass	NA	NA
Ignacio Sajoux et al. 2019 [51]	40 patients with overweight or obesity	VLCKD:47.1 ± 10.2; LCD:49.9 ± 9.3	Intervention Study	VLCKD:<10 g/d CHO, 10 g/d fat; LCD:40–55%CHO, 30%fat, 15–30%protein	2 months	body mass, fat mass, fat-free mass	NA	NA
Grant D Brinkworth et al. 2009(b) [52]	69 abdominal obesity adults with at least one metabolic risk factor	LCD:50.4 ± 8.1; LFD:49.7 ± 8.1	RCT	LCD:4%CHO, 61%fat, 35%protein; LFD:46%CHO, 30%fat, 24%protein	1 year	body mass, fat mass, fat-free mass	NA	NA
Pal T Jabekk et al. 2010 [53]	18 untrained women with BMI > 25 kg/m^2^	NA	RCT	Lc + Ex:6 ± 3%CHO, 66 ± 5%fat, 22 ± 4%protein; Ex:41 ± 4%CHO, 34 ± 3%fat, 17 ± 2%protein	10 weeks	body mass, fat mass, lean mass	NA	NA
Shengyan Sun et al. 2019 [54]	35 overweight or obese Chinese females	21.2 ± 3.3	RCT	LCD:9.3 ± 5.5%CHO, 68.1 ± 4.6%fat, 22.8 ± 3.2%protein; CD:43.1 ± 7.9%CHO, 40.2 ± 5.7%fat, 10.5 ± 3.6%protein	4 weeks	body mass	NA	VO_2_
Jeannie Tay et al. 2018 [55]	115 T2D patients with BMI of 26 to 45 kg/m^2^	58 ± 7	RCT	LC:14%CHO, 58%fat, 28%protein; HC:53%CHO, 30%fat, 17%protein	2 years	body mass, fat mass, fat-free mass	NA	NA
K. A. McAuley et al. 2005 [56]	58 women with BMI > 27 kg/m^2^	HFD:45 ± 7.4; HC:45 ± 7.5	RCT	HFD:26 ± 11%CHO, 47 ± 8%fat, 24 ± 6%protein; HC:45 ± 7%CHO, 28 ± 7%fat, 21 ± 3%protein	24 weeks	body mass, fat mass, fat-free mass	NA	NA
Carol S Johnston et al. 2006 [57]	19 adults with BMI > 25 kg/m^2^	KLC:38.4 ± 3.9; NLC:37.2 ± 3.9	RCT	KLC:9%CHO, 60%fat, 33%protein; NLC:42%CHO, 30%fat, 31%protein	2 weeks	body mass, fat mass, fat-free mass	NA	NA
Antonio Paoli et al. 2012 [58]	8 athletes, elite artistic gymnasts	20.9 ± 5.5	Non-randomized study	VLCKD:4.5 ± 0.5%CHO, 54.8 ± 6.0%fat, 40.7 ± 5.7%protein; WD:46.8 ± 2.1%CHO, 38.5 ± 2.6%fat, 14.7 ± 1.1%protein	30 days	body mass, fat mass, lean mass	squat jump, CMJ. reverse grip chins, push-ups, legs closed barrier, parallel bar dips	NA
Maryam Hadizadeh et al. 2020 [59]	20 untrained individuals	KD:35.2 ± 10.1; RD:33.2 ± 10.5	RCT	KD:5%CHO, 70%fat, 25%protein; RD:35%CHO, 20%fat, 45%protein	8 weeks	body mass, fat mass, lean mass	NA	NA
Vivian L Veum et al. 2017 [60]	38 with BMI > 29 kg/m^2^ and waist circumference > 98 cm	30–50	RCT	VHFLC:10%CHO, 73%fat, 17%protein; HCLF:53%CHO, 30%fat, 17%protein	12 weeks	body mass, fat mass, fat-free mass. skeletal muscle mass	NA	NA
Jeff S. Volek et al. 2002 [61]	20 men	LCD:36.7 ± 11.6; HD:35.0 ± 13.0	Intervention Study	LCD:8 ± 3%CHO, 61 ± 4%fat, 30 ± 5%protein; HD:58 ± 7%CHO, 26 ± 7%fat, 26 ± 2%protein	6 weeks	body mass, fat mass, lean mass	NA	NA
David M Shaw et al. 2019 [62]	8 trained male endurance athletes	29.6 ± 5.1	Randomized crossover study	HD:43%CHO, 38%fat; KD:4%CHO, 78%fat;	4 weeks	body mass	NA	VO_2_; RER; RPE

BMI: body mass index; CHO: carbohydrate; NA: not available; LCKD: low-carbohydrate ketogenic diet; UD: usual diet; CKD: cyclical ketogenic reduction diet; RD: regular diet; KD: ketogenic diet; WD: western diet; NKD: non-KD; CMJ: countermovement jump; BD: balanced nutrition drink; WD: western diet; HFD: high-fat diet; LFD: low-fat diet; LCHF: low carbohydrate high fat; HCLF: high carbohydrate low fat; VLCHF: very low-carbohydrate high-fat diet; HD: habitual diet; MD: mixed diet; HC: high-carbohydrate; LC: low-carbohydrate; LCHF: ketogenic low carbohydrate, high fat; HCHO: high CHO; CD: control diet; VLCKD: very low-calorie ketogenic diet; SHMD: standard hypocaloric Mediterranean diet; LCD: low-calorie diet; Ex: resistance exercise combined with a regular diet; Lc + Ex: resistance exercise in combination with a low carbohydrate ketogenic diet; KLC: ketogenic low-carbohydrate; NLC: nonketogenic low-carbohydrate; ACS: American Cancer Society diet; VHFLC: very high–fat, low-carbohydrate; VO_2_: oxygen consumption; VCO_2_, carbon dioxide production; RER: respiratory exchange ratio; TTE: treadmill time to exhaustion; RPE: rating of perceived exertion

### Assessment of publication bias and quality of evidence

The results of publication bias with fat-free mass (FFM) (*p* = 0.450) and fat mass (*p* = 0.103) indicated that there was no publication bias presented (Figure S1). Other indicators of the meta-analyses were limited by the fact that there were less than 10 studies included; hence, these were not assessable to mitigate potential publication bias. Quality assessment of 19 RCTs showed that 6 studies were considered “high risk” of bias due to blinding not implemented, imperfect allocation concealment, or incompletable outcome (Figure S2). Five crossover trials were evaluated using ROB 2, and one of them without a washout period was rated with a high risk of bias (Table S1). Among nine NRSI, one study was regarded as high risk for the baseline characteristics of study population mismatching (Table S2). Evidence assessment, according to the GRADE system, was evaluated in a range from moderate to very low (Table S3).

### Evidence synthesis

#### Muscle mass

**Fig. 2:**
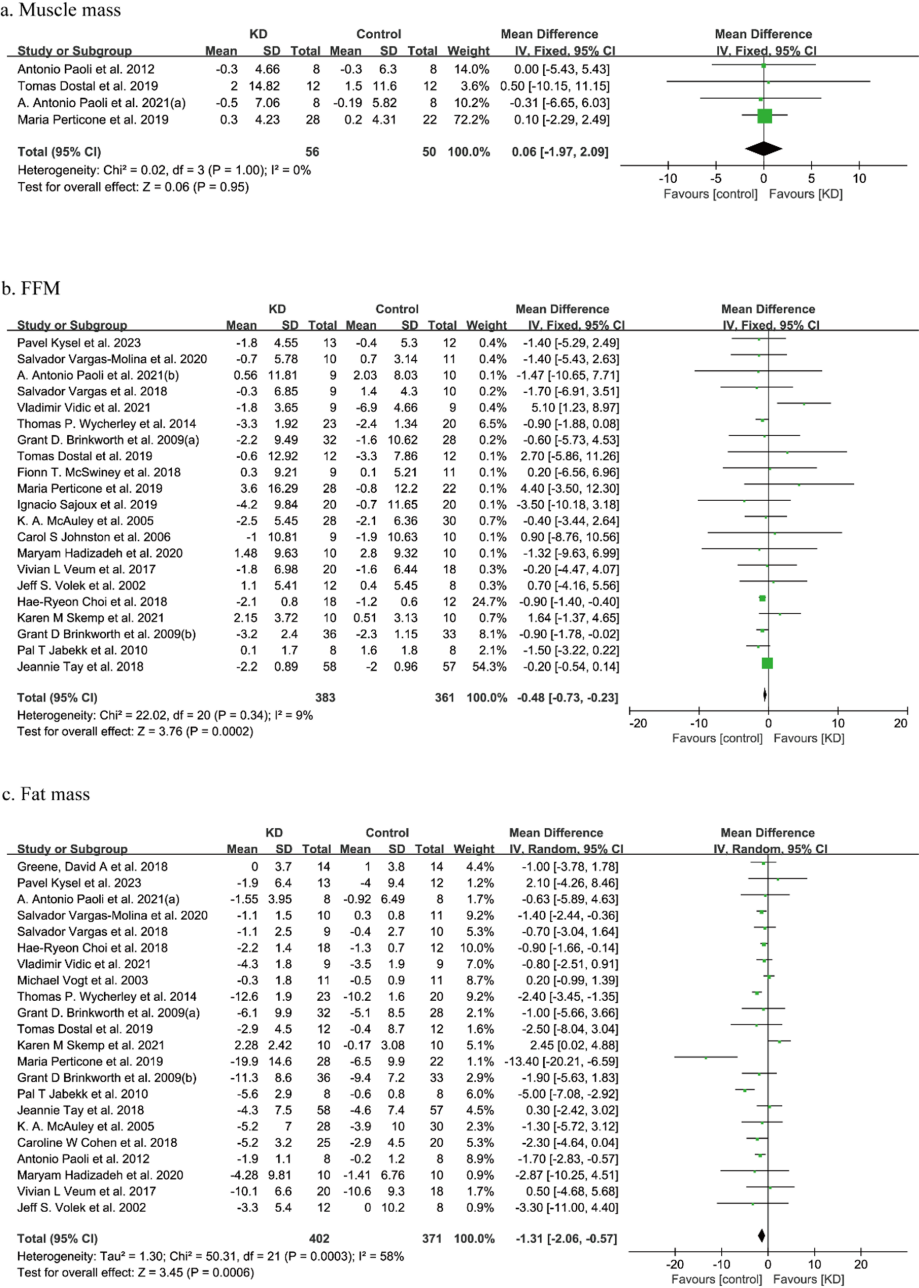
Forest plot for changes in muscle mass after the KD intervention. FFM: fat-free mass

Muscle mass was reported in four studies involving 106 participants. No significant difference in muscle mass was found between the KD and the control diet (WMD: 0.06, 95%CI: -1.97 to 2.09, *p* = 0.95). Through comprehensive analysis, low heterogeneity was found in this analysis (*I*^*2*^ = 0%) (Fig. 2a).

Except for muscle mass, FFM was also conducted as an alternative description of body muscle mass. There were 744 participants from 21 studies enrolled in the overall meta-analysis, and the result indicated a significant decrease in FFM with the KD versus the control diet (WMD: -0.48, 95%CI: -0.73 to -0.23, *p* < 0.001) (Fig. 2b). A small heterogeneity was detected in the meta-analysis (*I*^*2*^ = 9%). Based on the subgroup analysis, we did not find such effects in those performed on athletes, non-RCTs, and duration of intervention < 3months, despite a significant effect of KD on FFM in studies done on comprehensive analysis (Table S4).

Fat mass was assessed in 22 studies involving a total of 773 participants. The meta-analysis showed a significant reduction in fat mass in the KD group compared with the control diet (WMD: -1.31, 95%CI: -2.06 to -0.57, *p* < 0.001) (Fig. 2c). The analysis revealed moderate heterogeneity (*I²* = 58%). Subgroup analyses indicated that the reduction in fat mass remained significant in both RCTs and studies conducted in non-athlete populations (Table S4).

#### Muscle power and strength

##### CMJ

**Fig. 3:**
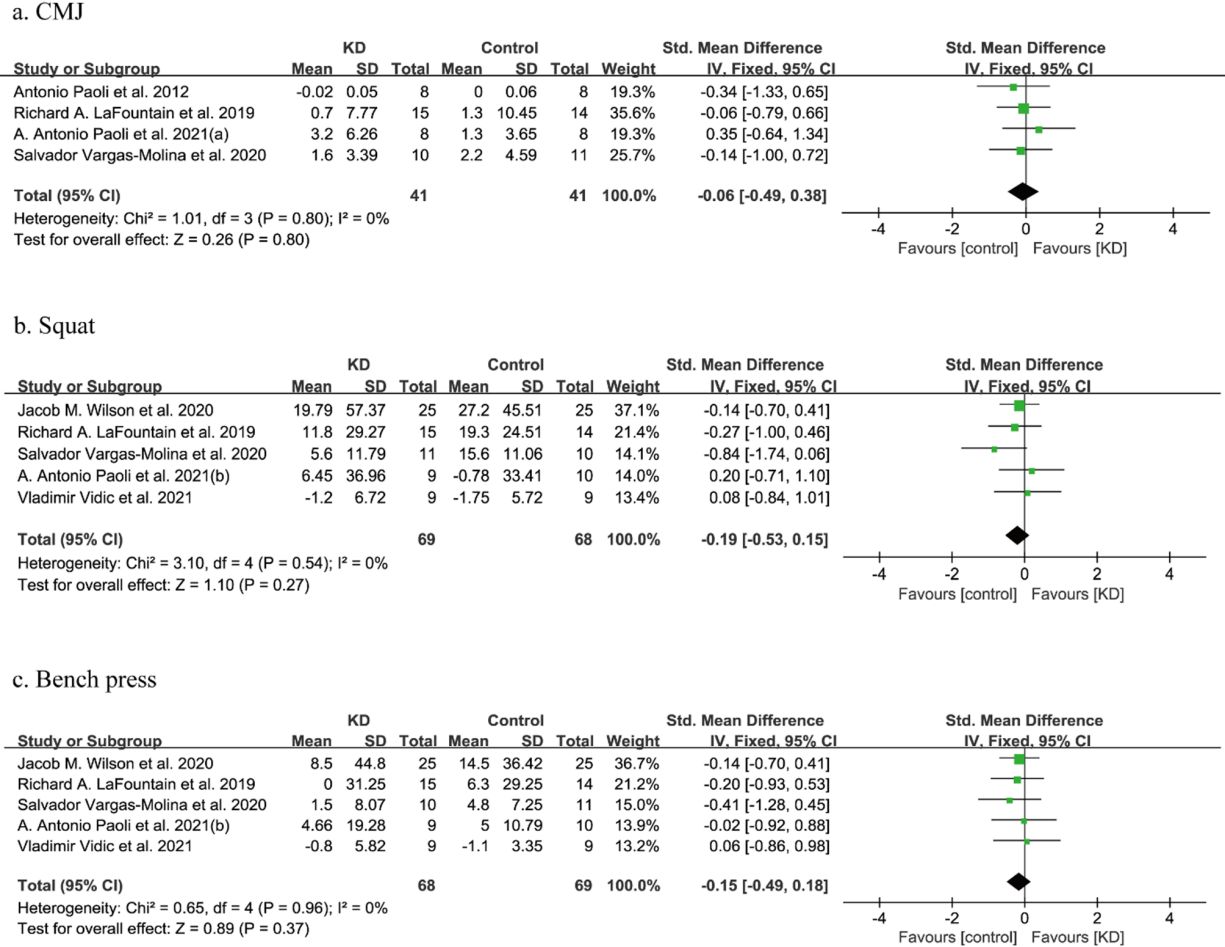
Forest plot for changes in muscle power and strength after the KD intervention. CMJ: countermovement jump

CMJ was recorded in four studies involving 82 individuals with a certain amount of training. The fixed-effect meta-analysis of the results indicated there was no significant difference in participants who ate a KD overall (SMD: -0.06, 95%CI: -0.49 to 0.38, *p* = 0.80). Tests for heterogeneity showed low statistical heterogeneity (*I*^*2*^ = 0%) (Fig. 3a).

##### Squat

The analysis included data from 137 individuals across five studies that measured squat weight. The meta-analysis for squat revealed no conclusive proof of variance between the two diets (SMD: -0.19, 95%CI: -0.53 to 0.15, *p* = 0.27). Heterogeneity was low (*I*^*2*^ = 0%) (Fig. 3b).

##### Bench press

Figure 3c shows the results of the connection of the KD versus other diets with bench press. The forest plot indicated that there was no statistically significant decline in the weight of bench press (SMD: -0.15, 95%CI: -0.49 to 0.18, *p* = 0.37) after the KD intervention. No heterogeneity was observed (*I*^*2*^ = 0%).

#### Aerobic metabolic capacity and endurance

##### VO_2max_ and VO_2max_ relative to body weight

**Fig. 4:**
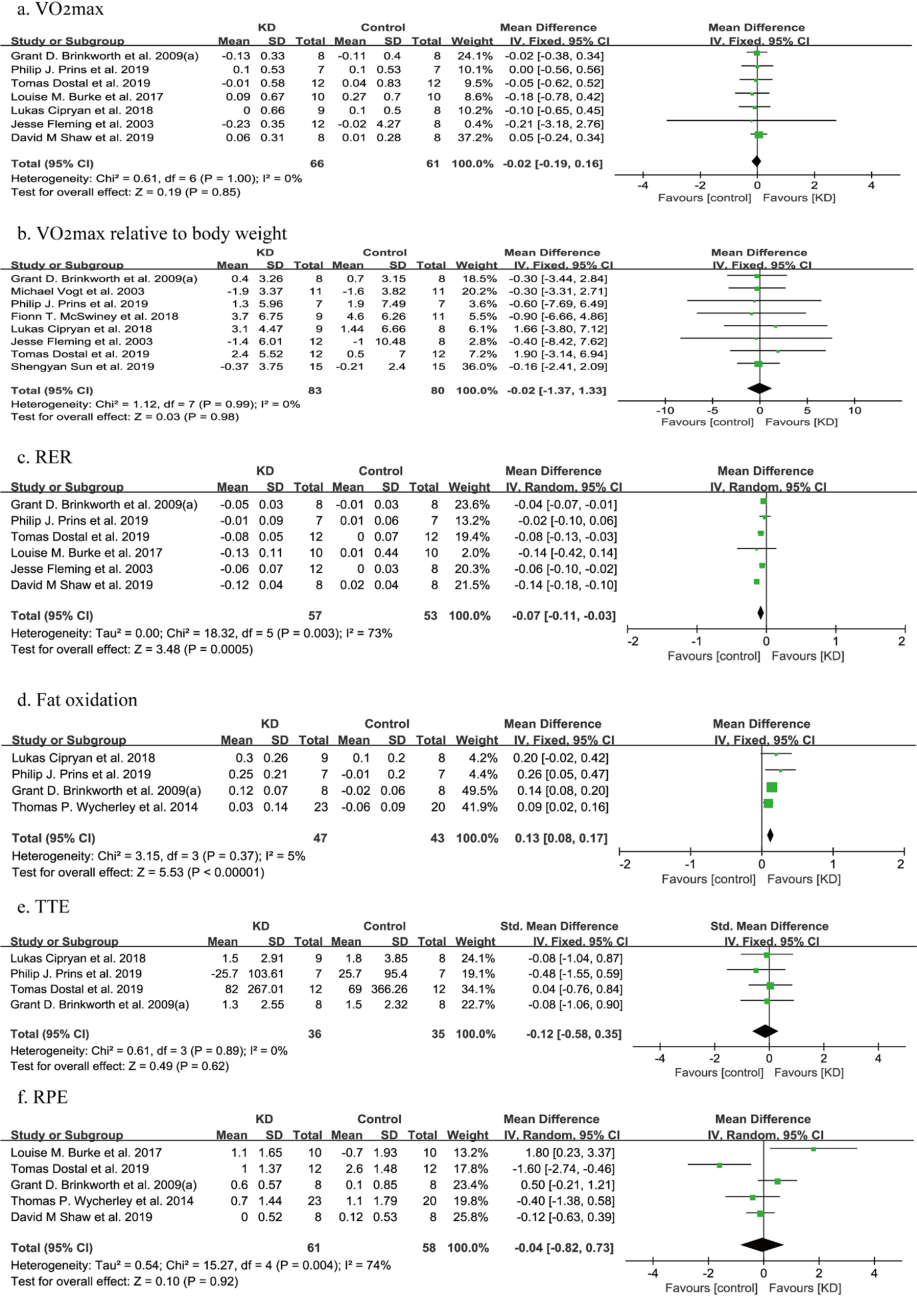
Forest plot for changes in aerobic metabolic capacity and endurance after the KD intervention. TTE: treadmill time to exhaustion; RPE: rating of perceived exertion; RER: respiratory exchange ratio

Figure 4a and b show separate meta-analyses of the associations of the KD versus the control diet with VO_2max_ and VO_2max_ relative to body weight. None of these differences were statistically significant (VO_2max_: WMD: -0.02, 95%CI: -0.19 to 0.16, *p* = 0.85; VO_2max_ relative to body weight: WMD: -0.02, 95%CI: -1.37 to 1.33, *p* = 0.98). The analysis of VO_2max_ enrolled 127 participants from 7 studies, and the result of VO_2max_ relative to body weight enrolled 163 participants from 8 studies respectively, with no heterogeneity existing (*I*^*2*^ = 0%).

##### Respiratory exchange ratio (RER)

Outcome data from 6 studies comprising 110 individuals were incorporated into the meta-analysis on RER. A notable decrease in RER was observed with the KD compared to the control group (WMD: -0.07, 95%CI: -0.11 to -0.03, *p* < 0.001). There was high heterogeneity based on the *I* [2] statistics (*I*^*2*^ = 73%) (Fig. 4c). And the subgroup analysis indicated that the effect of KD on RER was not statistically different in the subset of athletes (Table S4).

##### Fat oxidation

The fat oxidation was detailed in 4 studies, which included 90 participants. The KD can increase the level of fat oxidation (SMD: 0.13, 95%CI: 0.08 to 0.17, *p* < 0.001). In addition, high heterogeneity is observed in meta-analysis (*I*^*2*^ = 5%) (Fig. 4d).

##### Treadmill time to exhaustion (TTE)

Four studies were included in the meta-analysis of the data of TTE, constituting 71 participants. The tests for statistical heterogeneity indicated that heterogeneity was low (*I*^*2*^ = 0%). And the comprehensive analysis of results indicated that there was no evidence that the KD has an influence on the TTE (SMD: -0.12, 95%CI: -0.58 to 0.35, *p* = 0.62) (Fig. 4e).

##### Rating of perceived exertion (RPE)

There were 119 individuals from 5 studies reporting the change in RPE after the KD intervention. The results presented in Fig. 4f indicated no distinction in RPE between the KD and control diet groups (WMD: -0.04, 95%CI: -0.82 to 0.73, *p* = 0.92), existing large quantities of heterogeneity (*I*^*2*^ = 74%).

### Sensitivity analysis

Sensitivity analysis was carried out by systematically examining the impact of each individual study on the overall results of the meta-analysis. As Figure S3-S5 showed, the results of muscle mass, muscle strength, aerobic metabolic capacity and endurance were stable in meta-analysis. Removing any one of these studies did not influence the final conclusion of this study.

## Discussion

We investigated the effects of the KD on muscle outcomes, including muscle mass, muscle power and strength, aerobic metabolic capacity, and endurance. In the present study, we demonstrated that the KD intervention decreased FFM and fat mass, increased fat oxidation, and decreased RER during the treadmill test.

The study examined the impact of the ketogenic diet on muscle mass. Findings revealed that participants did not experience a decrease in muscle mass while following the KD, making it an effective method for fat loss. Although the heterogeneity of these analyses was low, the limited number of included studies may affect the validity of the results. Our study revealed a reduction in FFM with KD. While FFM is not equal to muscle mass, it does raise the concern that the KD may lead to a decrease in muscle mass. A previous study reported that KD could serve as an alternative approach to increase FFM in well-trained resistance athletes, particularly when applied in an energy-surplus context. However, due to the satiety-inducing nature of the diet and challenges with long-term adherence, it may not be the most effective strategy for promoting muscle hypertrophy in broader or less-trained populations [22]. Given the limited availability of muscle mass data in existing studies, further trials are necessary to validate this potential effect. Consistently, our analysis also demonstrated a significant decrease in fat mass with KD, confirming its efficacy in body fat reduction. This result aligns with previous studies highlighting KD’s lipolytic effects and metabolic benefit [4].

Muscle power and strength are essential components for evaluating muscular function and performance. From a physiological perspective, carbohydrates serve as the primary energy source during high-intensity and resistance exercises. Therefore, carbohydrate restriction inherent in KD protocols can reduce glycogen storage in skeletal muscle, potentially impairing performance in tasks that require rapid force generation or sustained power output [23]. However, evidence does not uniformly support this concern. A study by Vargas-Molina et al. investigated the effects of a ketogenic diet combined with resistance training on muscular strength in trained individuals. Their findings indicated that KD did not negatively affect muscle power and strength [22]. This is consistent with our meta-analysis, which found no significant differences in muscle power or strength between the KD and control diet groups.

Furthermore, aerobic metabolic capacity was detected in the analysis. Enhanced fat oxidation may result from the significant reduction in carbohydrate intake associated with the KD. The RER is defined as the ratio of carbon dioxide production to oxygen consumption. Since fat oxidation produces less carbon dioxide, an increase in fat oxidation leads to a decrease in the RER value [24]. Thus, the ketogenic diet gradually transitions the body from dependence on glucose to a reliance on fat and ketone bodies as the primary energy sources, resulting in increased fat oxidation and a reduction in RER values. VO_2max_, or maximum oxygen uptake, serves as an indicator of cardiopulmonary function. The intervention periods of the studies included in this analysis varied from 4 to 12 weeks, suggesting that short-term KD interventions are unlikely to significantly enhance cardiopulmonary function.

Further, the KD did not affect the TTE, and RPE values. The ketogenic diet significantly reduces carbohydrate intake, leading to diminished muscle glycogen stores. Since glycogen is a primary fuel source for high-intensity exercise, this could explain why TTE remains unchanged despite the increased reliance on fat as an energy source [25, 26]. Mechanistically, the KD would be expected to improve muscle performance due to the thermodynamic advantages of ketone oxidation [27]. However, the rate of energy provision from fat metabolism is slower compared to carbohydrate oxidation. This metabolic limitation might lead to a ceiling effect on TTE in activities requiring quick energy release. Adaptation to KD may lead to increased reliance on fat as a substrate during exercise, but this does not necessarily alter the central perception of effort, as the ketogenic state does not mitigate the neuromuscular fatigue associated with prolonged exercise [28]. Above all, ketone bodies were considered to provide more energy and increase energy efficiency, but there is no consistent evidence that the KD improves endurance performance [29, 30] which is consistent with the results of the meta-analysis conducted among endurance athletes [21]. Our comprehensive analysis included individuals with metabolic abnormalities (such as overweight, obesity, or risk factors for metabolic syndrome) as well as those engaged in strength training; thus, together, the results indicated that the KD did not improve exercise endurance or reduce fatigue. Of course, additional studies are needed to confirm the influence of the KD on time trial and sub-maximal endurance exercise performance.

Subgroup analyses suggest that longer KD interventions (≥ 3 months) are associated with more pronounced reductions in fat mass and fat-free mass, potentially reflecting cumulative effects of sustained carbohydrate restriction. In contrast, functional outcomes such as muscular strength, aerobic capacity, and endurance appeared unaffected by intervention length, indicating that metabolic adaptations alone may be insufficient to elicit measurable performance gains. Regarding methodological quality, RCTs yielded more consistent and statistically robust effects, particularly in FFM and fat mass, compared to non-randomized studies. The predominance of parallel-group designs, while appropriate for dietary comparisons, may be limited by inter-individual variability. Crossover trials may be limited by insufficient washout periods. These design-related factors likely contribute to the observed heterogeneity and should be addressed in future research planning.

This meta-analysis also has several limitations. To start with, the data of RPR, RER, and fat mass indicated the existence of heterogeneity in the included studies. Moreover, some included studies did not provide biochemical confirmation of ketosis (e.g., blood or urine ketone levels). Although we adopted the original studies’ definitions of KD, this may have introduced some bias, as not all low-carbohydrate diets necessarily induce ketosis. Furthermore, despite the FFM and fat mass, other pooled analyses only included several eligible studies and the data were chiefly comprised from single-center trials with small sample sizes. Nevertheless, sensitivity analysis validated the reliability of the results. Ultimately, the included studies were performed in different locations and evaluated with different equipment, which made it challenging to assess the potential bias.

In conclusion, there is no evidence yet that the KD may reduce muscle mass and muscle strength in our analysis. Moreover, we did find that the KD may influence the energy metabolism of muscles by enhancing fat oxidation. Given the restricted number of studies considered, additional research, particularly large multicenter RCTs, is required to confirm these findings.

## Supplementary Information

Below is the link to the electronic supplementary material.

Supplementary Material 1

## Data Availability

No datasets were generated or analysed during the current study.
