# Supplementary material for: Effects of ketogenic diet on muscle mass, strength, aerobic metabolic capacity, and endurance in adults: a systematic review and meta-analysis
Source: J Health Popul Nutr. 2025 Oct 1;44:346. doi: 10.1186/s41043-025-01090-z (PMC12487320; doi:10.1186/s41043-025-01090-z)
Supplement: Supplementary file 1 — Supplementary Material 1 [file 41043_2025_1090_MOESM1_ESM.docx]

SUPPLEMENTARY FILE

**Effects of ketogenic diet on muscle mass, strength, aerobic metabolic capacity, and endurance in adults: a systematic review and meta-analysis**

Yaqi Wang, Quanzhou Xiao, Zhenming Zhang, Yan Yang

Correspondence to: Yan Yang; National Clinical Research Center for Metabolic Diseases, Metabolic Syndrome Research Center, Key Laboratory of Diabetes Immunology (Central South University), Ministry of Education, and Department of Metabolism and Endocrinology, The Second Xiangya Hospital of Central South University, Changsha 410011, Hunan, China; Tel: +86 17307418036 E-mail: 228201015@csu.edu.cn

**Figure S1.** Funnel plot of publication bias

**Figure S2.** Publication bias of RCTs included in the meta-analysis

**Figure S3.** Sensitively analysis of muscle mass

**Figure S4.** Sensitively analysis of muscle power and strength

**Figure S5.** Sensitively analysis of aerobic metabolic capacity and endurance

**Table S1.** Quality analysis of crossover trials included in the meta-analysis

**Table S2.** Quality analysis of NRSI included in the meta-analysis

**Table S3.** Evidence profile assessment according to GRADE framework

**Table S4**. Subgroup analysis of included studies in meta-analysis of the effects of KD on muscle outcomes

**Figure S1. Funnel plot of publication bias**


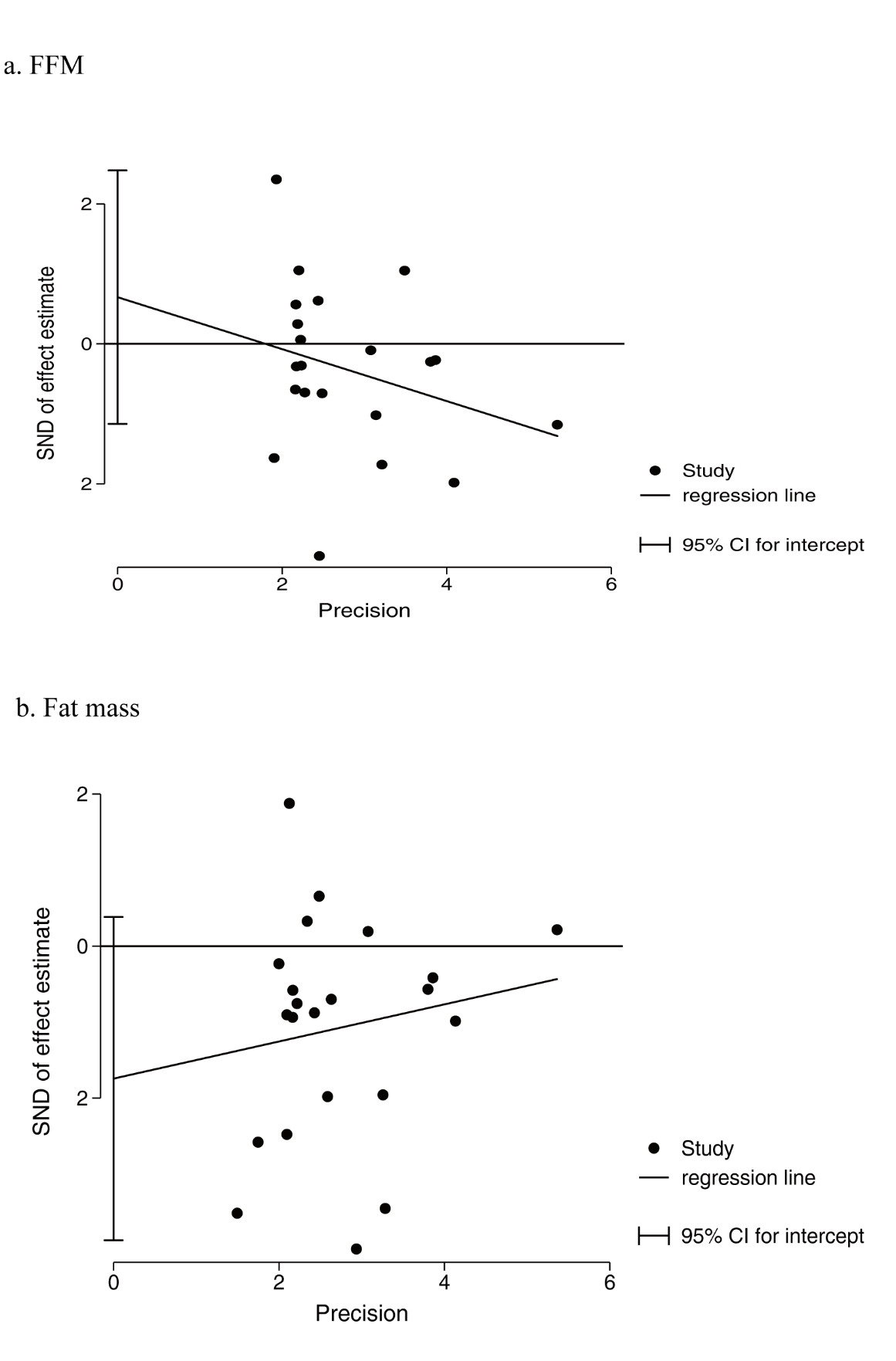


FFM: fat-free mass.

**Figure S2. Publication bias of RCTs included in the meta-analysis**
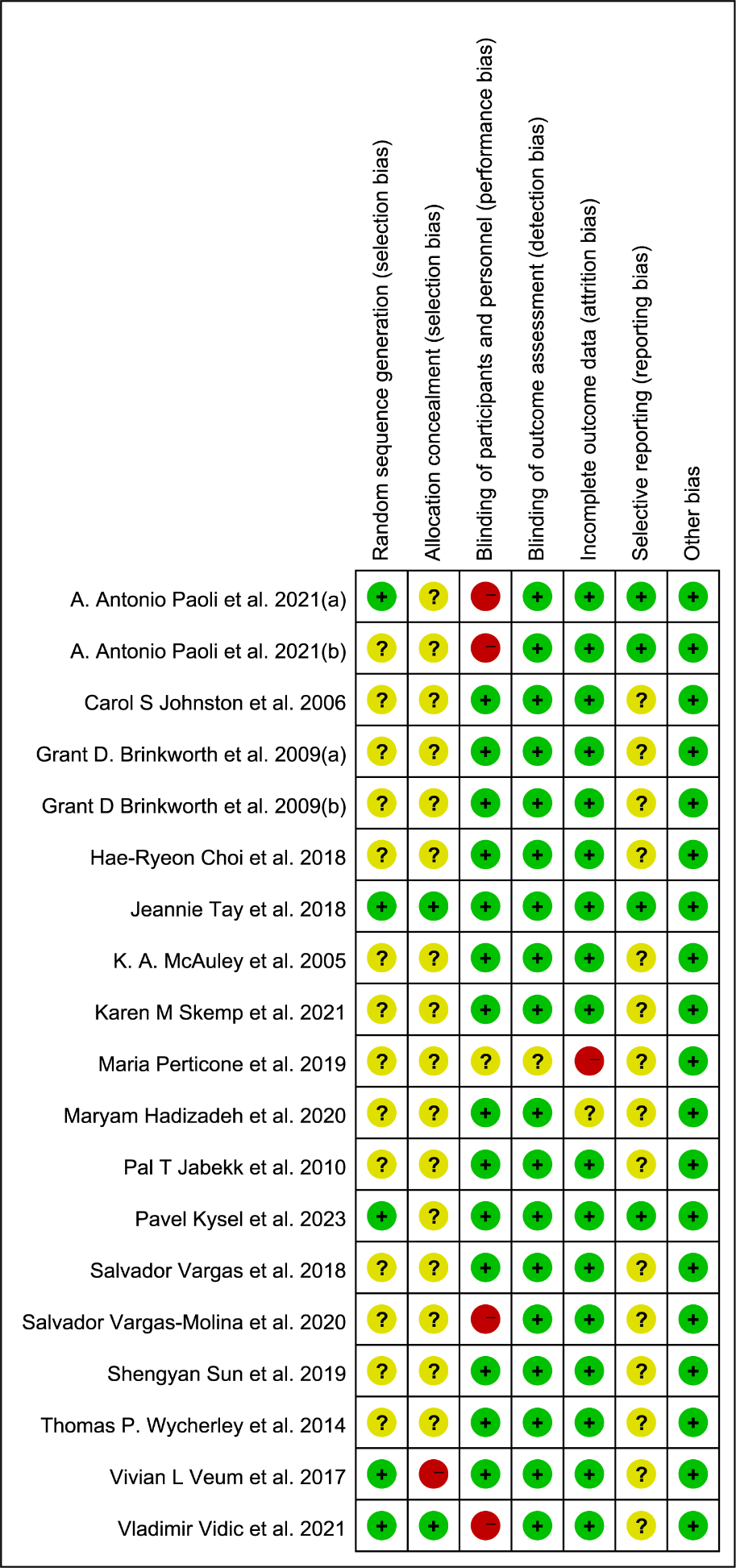


**Figure S3. Sensitively analysis of muscle mass**


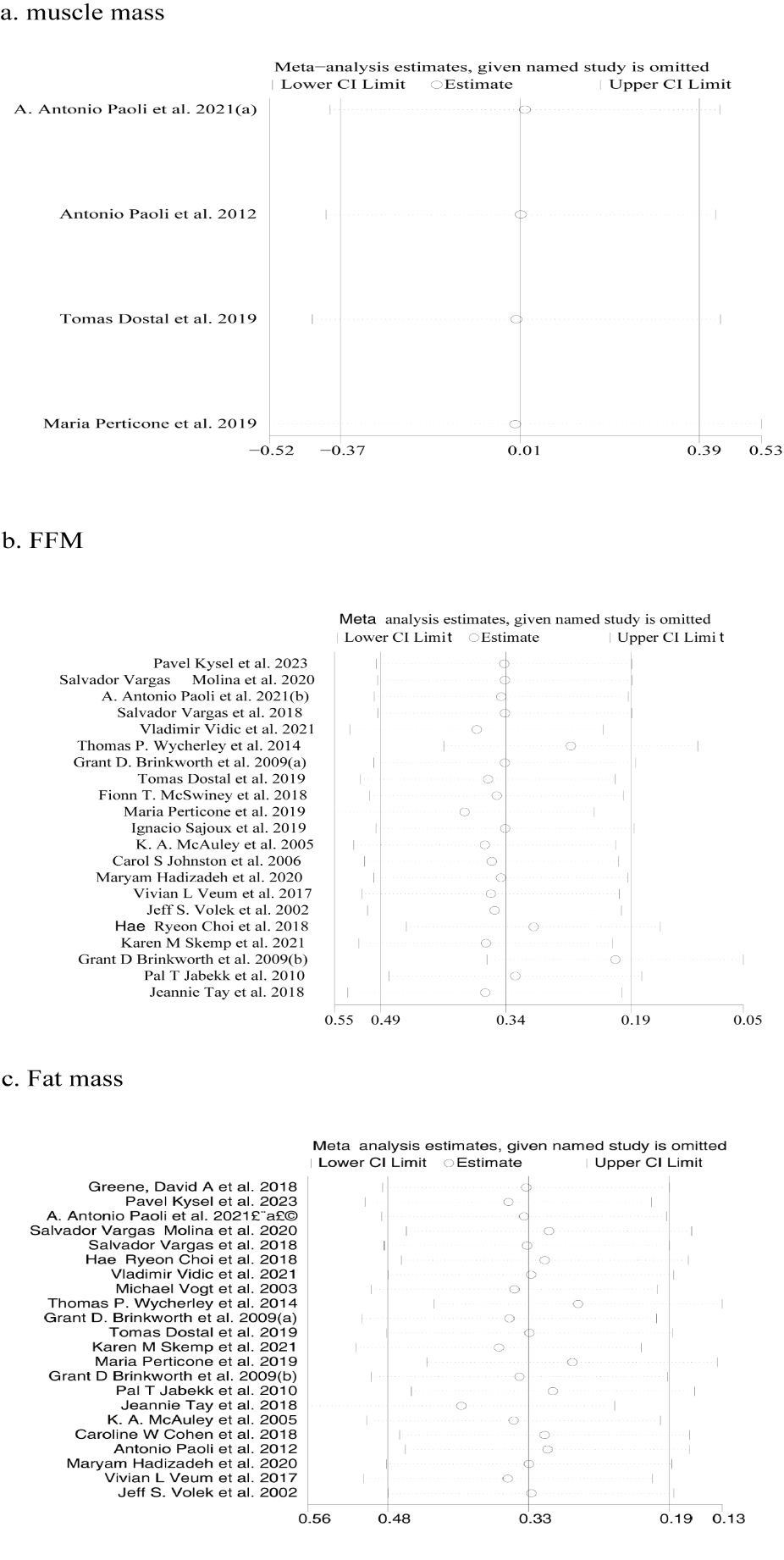


FFM: fat-free mass.

**Figure S4. Sensitively analysis of muscle power and strength**


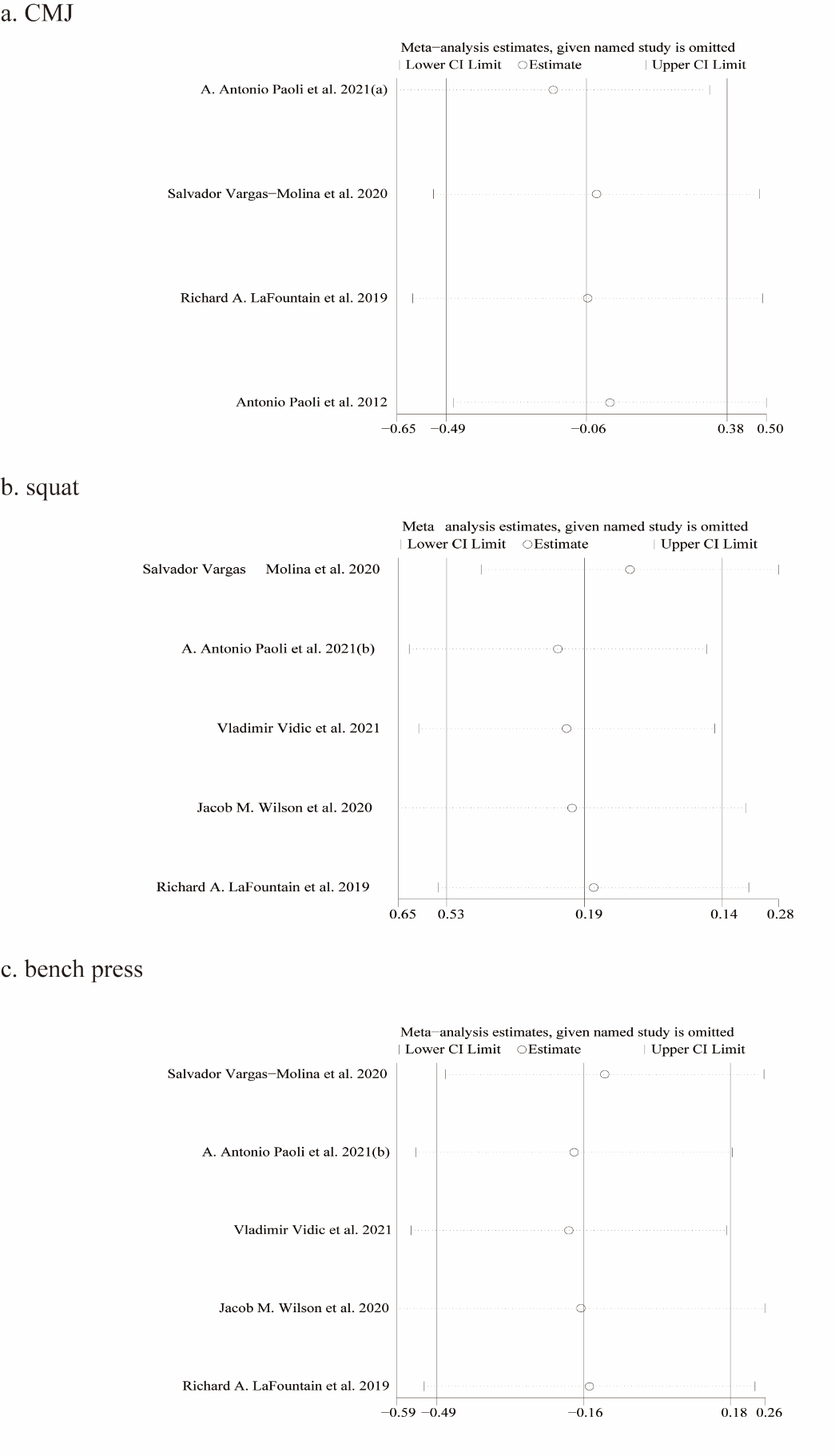


CMJ: countermovement jump.

**
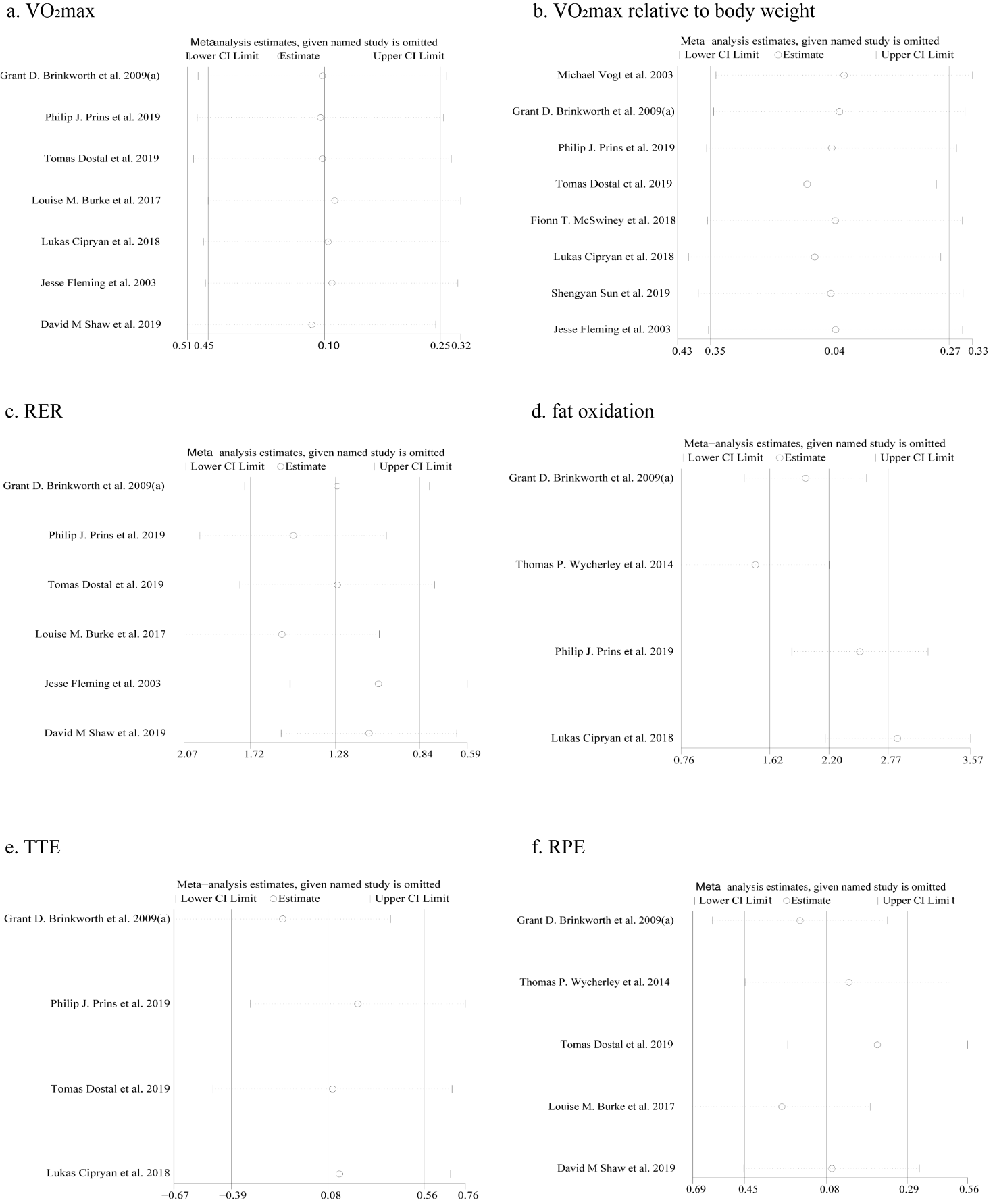
Figure S5. Sensitively analysis of aerobic metabolic capacity and endurance**

RER: respiratory exchange ratio; TTE: treadmill time to exhaustion; RPE: rating of perceived exertion.

**Table S1. Quality analysis of crossover trials included in the meta-analysis**

| **Study ID**  **Type of bias** | Greene, David A et al. 2018 | Jacob M. Wilson et al. 2020 | Michael Vogt et al. 2003 | Philip J. Prins et al. 2019 | David M Shaw et al. 2019 |
| --- | --- | --- | --- | --- | --- |
| Randomization process | some concerns | some concerns | some concerns | some concerns | some concerns |
| Risk of bias arising from period and carryover effects | low risk | low risk | high risk | low risk | low risk |
| Deviations from intended interventions | low risk | low risk | low risk | low risk | low risk |
| Mising outcome data | low risk | low risk | low risk | low risk | low risk |
| Measurement of the outcome | low risk | low risk | low risk | low risk | low risk |
| Selection of the reported result | low risk | some concerns | some concerns | some concerns | low risk |
| Overall Bias | some concerns | some concerns | high risk | some concerns | some concerns |

**Table S2. Quality analysis of NRSI included in the meta-analysis**

| **Study ID**  **Type of bias** | **Tomas Dostal et al. 2019** | **Richard A. LaFountain et al. 2019** | **Fionn T. McSwiney et al. 2018** | **Louise M. Burke et al. 2017** | **Lukas Cipryan et al. 2018** | **Jesse Fleming et al. 2003** | **Ignacio Sajoux et al. 2019** | **Antonio Paoli et al. 2012** | **Jeff S. Volek et al. 2002** |
| --- | --- | --- | --- | --- | --- | --- | --- | --- | --- |
| Bias due to confounding | low | moderate | moderate | moderate | low | low | moderate | low | moderate |
| Bias in selection of participants into the study | low | low | low | moderate | low | low | low | low | low |
| Bias in classification of interventions | low | low | low | low | low | low | low | low | low |
| Bias due to deviations from intended interventions | low | serious | moderate | low | low | low | moderate | low | low |
| Bias due to missing data | low | low | low | low | low | low | moderate | low | low |
| Bias in measurement of outcomes | low | low | low | low | low | low | low | low | low |
| Bias in selection of the reported result | low | moderate | moderate | low | low | low | low | low | low |

**Table S3.** **Evidence profile assessment according to GRADE framework**

| **Outcomes** | **Illustrative comparative risks* (95% CI)** | | **Relative effect (95% CI)** | **No of Participants (studies)** | **Quality of the evidence (GRADE)** | |
| --- | --- | --- | --- | --- | --- | --- |
|  | Assumed risk | Corresponding risk |  |  |  |  |
|  | **Control** | **KD** |  |  |  |  |
| **muscle mass for randomised trials** |  | The mean muscle mass for randomised trials in the intervention groups was **0.05 higher** (2.18 lower to 2.28 higher) |  | 66 (2 studies) | ⊕⊕⊝⊝ **low**^1,2^ | |
| **muscle mass for NRSI** |  | The mean muscle mass for nrsi in the intervention groups was **0.10 higher** (4.74 lower to 4.94 higher) |  | 40 (2 studies) | ⊕⊝⊝⊝ **very low**^2^ | |
| **FFM for randomised trials** |  | The mean ffm for randomised trials in the intervention groups was **0.57 lower** (1.00 lower to 0.15 lower) |  | 640 (17 studies) | ⊕⊕⊝⊝ **low**^1,3^ | |
| **FFM for NRSI** |  | The mean ffm for nrsi in the intervention groups was **0.07 lower** (3.23 lower to 3.08 higher) |  | 104 (4 studies) | ⊕⊝⊝⊝ **very low**^2^ | |
| **Fat mass for randomised trials** |  | The mean fat mass for randomised trials in the intervention groups was **1.41 lower** (2.34 lower to 0.48 lower) |  | 663 (17 studies) | ⊕⊕⊝⊝ **low**^1,3^ | |
| **Fat mass for NRSI** |  | The mean fat mass for nrsi in the intervention groups was **0.93 lower** (2.07 lower to 0.22 higher) |  | 110 (5 studies) | ⊕⊝⊝⊝ **very low**^2^ | |
| **Counter movement jump for randomised trials** |  | The mean counter movement jump for randomised trials in the intervention groups was **0.07 standard deviations higher** (0.58 lower to 0.72 higher) |  | 37 (2 studies) | ⊕⊕⊝⊝ **low**^1,2^ | |
| **Counter movement jump for NRSI** |  | The mean counter movement jump for nrsi in the intervention groups was **0.16 standard deviations lower** (0.75 lower to 0.43 higher) |  | 45 (2 studies) | ⊕⊝⊝⊝ **very low**^2,4^ | |
| **Squat for randomised trials** |  | The mean squat for randomised trials in the intervention groups was **0.19 standard deviations lower** (0.72 lower to 0.33 higher) |  | 108 (4 studies) | ⊕⊕⊝⊝ **low**^1,2^ | |
| **Squat for NRSI** |  | The mean squat for nrsi in the intervention groups was **0.19 standard deviations lower** (0.69 lower to 0.25 higher) |  | 29 (1 study) | ⊕⊝⊝⊝ **very low**^2.4^ | |
| **Bench press for randomised trials** |  | The mean bench press for randomised trials in the intervention groups was **0.14 standard deviations lower** (0.52 lower to 0.24 higher) |  | 108 (4 studies) | ⊕⊕⊝⊝ **low**^1,2^ | |
| **Bench press for NRSI** |  | The mean bench press for nrsi in the intervention groups was **0.2 standard deviations lower** (0.593 lower to 0.53 higher) |  | 29 (1 study) | ⊕⊝⊝⊝ **very low**^2,4^ | |
| **VO_2_max for randomised trials** |  | The mean vo2max for randomised trials in the intervention groups was **0.02 lower** (0.15 lower to 0.11 higher) |  | 16 (1 study) | ⊕⊕⊕⊝ **moderate**^2^ | |
| **VO_2_max for NRSI** |  | The mean vo2max for nrsi in the intervention groups was **0.02 lower** (0.22 lower to 0.19 higher) |  | 111 (6 studies) | ⊕⊝⊝⊝ **very low**^2^ | |
| **VO_2_max relative to body weight for randomised trials** |  | The mean vo2 relative to body weight for randomised trials in the intervention groups was **0.21 lower** (2.04 lower to 1.62 higher) |  | 82 (4 studies) | ⊕⊕⊕⊝ **moderate**^2^ | |
| **VO_2_max relative to body weight for NRSI** |  | The mean vo2 relative to body weight for nrsi in the intervention groups was **0.21 higher** (1.80 lower to 2.21 higher) |  | 81 (4 studies) | ⊕⊝⊝⊝ **very low**^2^ | |
| **Treadmill time to exhaustion for randomised trials** |  | The mean treadmill time to exhaustion for randomised trials in the intervention groups was **0.08 standard deviations lower** (1.06 lower to 0.90 higher) |  | 30 (2 studies) | ⊕⊕⊕⊝ **moderate**^2^ | |
| **Treadmill time to exhaustion for NRSI** |  | The mean treadmill time to exhaustion for nrsi in the intervention groups was **0.13 standard deviations lower** (0.66 lower to 0.40 higher) |  | 41 (2 studies) | ⊕⊝⊝⊝ **very low**^2^ | |
| **Rating of perceived exertion for randomised trials** |  | The mean peak rating of perceived exertion for randomised trials in the intervention groups was **0.12 higher** (0.76 lower to 0.99 higher) |  | 59 (2 studies) | ⊕⊕⊝⊝ **low**^2.3^ | |
| **Rating of perceived exertion for NRSI** |  | The mean peak rating of perceived exertion for nrsi in the intervention groups was **0.07 lower** (1.54 lower to 1.40 higher) |  | 60 (3 studies) | ⊕⊝⊝⊝ **very low**^2.3^ | |
| **Respiratory exchange ratio for randomised trials** |  | The mean peak respiratory exchange ratio for randomised trials in the intervention groups was **0.04 lower** (0.07 lower to 0.01 lower) |  | 16 (1 studies) | ⊕⊕⊕⊝ **moderate**^2^ | |
| **Respiratory exchange ratio for NRSI** |  | The mean peak respiratory exchange ratio for nrsi in the intervention groups was **0.08 lower** (0.13 lower to 0.04 lower) |  | 94 (5 studies) | ⊕⊝⊝⊝ **very low**^2^ | |
| **Fat oxidation for randomised trials** |  | The mean fat oxidation for randomised trials in the intervention groups was **0.12 higher** (0.07 higher to 0.17 higher) |  | 73 (3 studies) | ⊕⊕⊕⊝ **moderate**^2^ | |
| **Fat oxidation for NRSI** |  | The mean fat oxidation for nrsi in the intervention groups was **0.23 higher** (0.08 lower to 0.38 higher) |  | 17 (1 study) | ⊕⊝⊝⊝ **very low**^2^ | |
| *The basis for the **assumed risk** (e.g. the median control group risk across studies) is provided in footnotes. The **corresponding risk** (and its 95% confidence interval) is based on the assumed risk in the comparison group and the **relative effect** of the intervention (and its 95% CI).  **CI:** Confidence interval; | | | | | | |
| GRADE Working Group grades of evidence **High quality:** Further research is very unlikely to change our confidence in the estimate of effect.  **Moderate quality:** Further research is likely to have an important impact on our confidence in the estimate of effect and may change the estimate. **Low quality:** Further research is very likely to have an important impact on our confidence in the estimate of effect and is likely to change the estimate. **Very low quality:** We are very uncertain about the estimate. | | | | | | |
| ^1^ Blinding not implemented ^2^The number of observational populations was limited  ^3^The results were not consistent  ^4^ Baseline data were not matched | | | | | | |

**Table S4. Subgroup analysis of included studies in meta-analysis of the effects of KD on muscle outcomes**

| **Items** | **Subgroup** | **No. of studies** | **WMD/SMD (95%CI)** | ***P* value** | ***I^2^* (%)** | ***P*-heterogeneity** | ***P* for between subgroup heterogeneity** |
| --- | --- | --- | --- | --- | --- | --- | --- |
| Muscle mass | Subjects |  |  |  |  |  | 0.92 |
|  | Athletes | 2 | -0.13 (-4.26, 3.99) | 0.95 | 0 | 0.94 |  |
|  | Non- athlete | 2 | 0.12 (-2.21, 2.45) | 0.92 | 0 | 0.94 |  |
|  | Study type |  |  |  |  |  | 0.98 |
|  | RCT | 2 | 0.05 (-2.18, 2.28) | 0.97 | 0 | 0.91 |  |
|  | Non-RCT | 2 | 0.10 (-4.73,4.94) | 0.97 | 0 | 0.93 |  |
|  | Duration of intervention |  |  |  |  |  | 0.92 |
|  | ≥ 3months | 2 | 0.12 (-2.21, 2.45) | 0.92 | 0 | 0.94 |  |
|  | < 3months | 2 | -0.13 (-4.26, 3.99) | 0.95 | 0 | 0.94 |  |
| FFM | Subjects |  |  |  |  |  | 0.95 |
|  | Athletes | 2 | -0.39 (-5.83, 5.06) | 0.89 | 0 | 0.77 |  |
|  | Non- athlete | 19 | -0.57 (-0.97, -0.16) | 0.006 | 18 | 0.23 |  |
|  | Study type |  |  |  |  |  | 0.76 |
|  | RCT | 17 | -0.57 (-1.00, -0.15) | 0.007 | 22 | 0.20 |  |
|  | Non-RCT | 4 | -0.07 (-3.23, 3.08) | 0.96 | 0 | 0.68 |  |
|  | Duration of intervention |  |  |  |  |  |  |
|  | ≥ 3months | 8 | -0.80 (-1.30, -0.29) | 0.002 | 5 | 0.39 | 0.32 |
|  | < 3months | 13 | -0.29 (-0.60, 0.01) | 0.06 | 0 | 0.84 |  |
| Fat mass | Subjects |  |  |  |  |  |  |
|  | Athletes | 4 | -0.80 (-2.00, 0.41) | 0.19 | 42 | 0.16 | 0.37 |
|  | Non- athlete | 18 | -1.48 (-2.40, -0.57) | 0.002 | 61 | <0.001 |  |
|  | Study type |  |  |  |  |  |  |
|  | RCT | 17 | -1.41 (-2.34, -0.48) | 0.003 | 63 | <0.001 | 0.52 |
|  | Non-RCT | 5 | -0.93 (-2.07, 0.22) | 0.11 | 32 | 0.21 |  |
|  | Duration of intervention |  |  |  |  |  | 0.28 |
|  | ≥ 3months | 9 | -1.94 (-3.39, -0.50) | 0.008 | 49 | 0.05 |  |
|  | < 3months | 13 | -1.02 (-1.88, -0.16) | 0.02 | 59 | 0.003 |  |
| CMJ | Subjects |  |  |  |  |  | 0.83 |
|  | Athletes | 2 | 0.00 (-0.70, 0.70) | 0.99 | 0 | 0.33 |  |
|  | Non- athlete | 2 | -0.10 (-0.49, 0.38) | 0.73 | 0 | 0.89 |  |
|  | Study type |  |  |  |  |  | 0.60 |
|  | RCT | 2 | 0.07 (-0.58, 0.72) | 0.83 | 0 | 0.46 |  |
|  | Non-RCT | 2 | -0.16 (-0.75, 0.43) | 0.59 | 0 | 0.66 |  |
|  | Duration of intervention |  |  |  |  |  | 0.98 |
|  | ≥ 3months | 1 | -0.06 (-0.79, 0.66) | 0.86 | NA | NA |  |
|  | < 3months | 3 | -0.16 (-0.75, 0.43) | 0.84 | 0 | 0.60 |  |
| Squat | Subjects |  |  |  |  |  | 0.37 |
|  | Athletes | 1 | 0.20 (-0.71, 1.10) | 0.67 | NA | NA |  |
|  | Non- athlete | 4 | -0.25 (-0.62, 0.11) | 0.18 | 0 | 0.51 |  |
|  | Study type |  |  |  |  |  | 0.99 |
|  | RCT | 3 | -0.19 (-0.72, 0.33) | 0.48 | 334 | 0.22 |  |
|  | Non-RCT | 2 | -0.19 (-0.63, 0.25) | 0.41 | 0 | 0.78 |  |
|  | Duration of intervention |  |  |  |  |  | 0.81 |
|  | ≥ 3months | 1 | -0.27 (-1.00, 0.46) | 0.47 | NA | NA |  |
|  | < 3months | 4 | -0.17 (-0.55, 0.21) | 0.39 | 1 | 0.38 |  |
| Brench press | Subjects |  |  |  |  |  | 0.76 |
|  | Athletes | 1 | -0.02 (-0.92, 0.88) | 0.96 | NA | NA |  |
|  | Non- athlete | 4 | -0.17 (-0.54, 0.18) | 0.35 | 0 | 0.91 |  |
|  | Study type |  |  |  |  |  | 0.93 |
|  | RCT | 3 | -0.14 (-0.65, 0.38) | 0.61 | 0 | 0.73 |  |
|  | Non-RCT | 2 | -0.17 (-0.61, 0.28) | 0.46 | 0 | 0.90 |  |
|  | Duration of intervention |  |  |  |  |  | 0.88 |
|  | ≥ 3months | 1 | -0.20 (-0.93, 0.53) | 0.59 | NA | NA |  |
|  | < 3months | 4 | -0.14 (-0.52, 0.24) | 0.47 | 0 | 0.89 |  |
| VO_2max_ | Subjects |  |  |  |  |  | 0.77 |
|  | Athletes | 3 | 0.01 (-0.23, 0.24) | 0.96 | 0 | 0.80 |  |
|  | Non- athlete | 4 | -0.05 (-0.31, 0.22) | 0.73 | 0 | 1.00 |  |
|  | Study type |  |  |  |  |  | 0.89 |
|  | RCT | 1 | -0.02 (-0.38, 0.34) | 0.91 | NA | NA |  |
|  | Non-RCT | 6 | -0.02 (-0.22, 0.19) | 0.87 | 0 | 0.99 |  |
|  | Duration of intervention |  |  |  |  |  | 0.91 |
|  | ≥ 3months | 1 | -0.05 (-0.62, 0.52) | 0.86 | NA | NA |  |
|  | < 3months | 6 | -0.01 (-0.20, 0.17) | 0.88 | 0 | 0.99 |  |
| VO_2max_ relative to body | Subjects |  |  |  |  |  | 0.69 |
|  | Athletes | 3 | -0.45 (-2.95, 2.05) | 0.72 | 0 | 0.98 |  |
|  | Non- athlete | 5 | 0.16 (-1.45, 1.77) | 0.84 | 0 | 0.92 |  |
|  | Study type |  |  |  |  |  | 0.76 |
|  | RCT | 2 | -0.21 (-2.04, 1.62) | 0.82 | 0 | 0.94 |  |
|  | Non-RCT | 6 | 0.21 (-1.80, 2.21) | 0.84 | 0 | 0.96 |  |
|  | Duration of intervention |  |  |  |  |  | 0.63 |
|  | ≥ 3months | 2 | 0.68 (-3.11, 4.48) | 0.72 | 0 | 0.47 |  |
|  | < 3months | 6 | -0.27 (-0.91, 0.37) | 0.41 | 0 | 0.99 |  |
| RER | Subjects |  |  |  |  |  | 0.45 |
|  | Athletes | 3 | -0.09 (-0.19, 0.01)  03 | 0.07 | 71 | 0.03 |  |
|  | Non- athlete | 3 | -0.05 (-0.08, -0.03) | <0.001 | 1 | 0.36 |  |
|  | Study type |  |  |  |  |  | 0.12 |
|  | RCT | 1 | -0.04 (-0.07, -0.01) | 0.008 | NA | NA |  |
|  | Non-RCT | 5 | -0.08 (-0.13, -0.04) | <0.001 | 0.02 | 0.64 |  |
|  | Duration of intervention |  |  |  |  |  | 0.93 |
|  | ≥ 3months | 1 | -0.08 (-0.13, -0.03) | 0.001 | NA | NA |  |
|  | < 3months | 5 | -0.08 (-0.13, -0.04) | <0.001 | 64 | 0.02 |  |
| Fat oxidation | Subjects |  |  |  |  |  | 0.21 |
|  | Athletes | 1 | 0.26 (0.05, 0.47) | 0.02 | NA | NA |  |
|  | Non- athlete | 3 | 0.12 (0.07, 0.17) | <0.001 | 0 | 0.45 |  |
|  | Study type |  |  |  |  |  | 0.17 |
|  | RCT | 2 | 0.12 (0.07, 0.17) | <0.001 | 7 | 0.30 |  |
|  | Non-RCT | 2 | 0.23 (0.08, 0.38) | 0.003 | 0 | 0.70 |  |
|  | Duration of intervention |  |  |  |  |  | 0.17 |
|  | ≥ 3months | 1 | 0.09 (0.02, 0.16) | 0.01 | NA | NA |  |
|  | < 3months | 3 | 0.15 (0.09, 0.21) | <0.001 | 0 | 0.53 |  |
| TTE | Subjects |  |  |  |  |  |  |
|  | Athletes | 1 | -0.48 (-1.55, 0.59) | 0.38 | NA | NA |  |
|  | Non- athlete | 3 | -0.03 (-0.55, 0.49) | 0.91 | 0 | 0.98 |  |
|  | Study type |  |  |  |  |  | 0.93 |
|  | RCT | 1 | -0.08 (-1.06, 0.90) | 0.88 | NA | NA |  |
|  | Non-RCT | 3 | -0.13 (-0.66, 0.40) | 0.64 | 0 | 0.74 |  |
|  | Duration of intervention |  |  |  |  |  | 0.64 |
|  | ≥ 3months | 1 | 0.04 (-0.76, 0.84) | 0.92 | NA | NA |  |
|  | < 3months | 3 | -0.20 (-0.77, 0.38) | 0.50 | 0 | 0.82 |  |
| RPE | Subjects |  |  |  |  |  | 0.32 |
|  | Athletes | 2 | 0.69 (-1.17, 2.55) | 0.47 | 81 | 0.02 |  |
|  | Non- athlete | 3 | -0.43 (-1.62, 0.75)  0.445 | 0.47 | 79 | 0.008 |  |
|  | Study type |  |  |  |  |  | 0.83 |
|  | RCT | 2 | 0.12 (-0.76, 0.99) | 0.79 | 53 | 0.14 |  |
|  | Non-RCT | 3 | 0.07 (-1.54, 1.40) | 0.92 | 83  33 | 0.002 |  |
|  | Duration of intervention |  |  |  |  |  | 0.05 |
|  | ≥ 3months | 2 | -0.96 (-2.14, 0.21) | 0.11 | 59 | 0.12 |  |
|  | < 3months | 3 | 0.46 (-0.36, 1.29) | 0.27 | 67 | 0.05 |  |

FFM: fat-free mass; CMJ: countermovement jump; TTE: treadmill time to exhaustion; RPE: rating of perceived exertion; RER: respiratory exchange ratio.
